# A Rare Case of Adult Medulloblastoma with Spinal Metastasis

**DOI:** 10.1155/2012/748601

**Published:** 2012-09-30

**Authors:** K. Quenum, J. Ntalaja, J. Onen, Y. Arkha, S. Derraz, A. El Ouahabi, S. Sefiani, A. ElKhamlichi

**Affiliations:** ^1^Department of Neurosurgery, Hôpital des Spécialités, Rabat, Morocco; ^2^Department of Pathology, Hôpital des Spécialités, Rabat, Morocco

## Abstract

Medulloblastoma is a relatively common malignant brain tumor of childhood and relatively rare in adulthood, with a propensity for neuraxial spread via cerebrospinal fluid pathways. Osseous extraneural metastasis is uncommon and when it happens, radiologic findings are of sclerotic (60%), lytic (35%), and mixed patterns (5%) (Algra et al. (1992)). In this paper, we present a case of medulloblastoma metastiaszing to the lumbar spine and describe the magnetic resonance appearance, with emphasis on the image findings mimicking spondylodiscitis.

## 1. Introduction

Due to advances in surgery, chemotherapy, and radiotherapy and differences between patients with localised and metastatic diseases, the prognosis of medulloblastoma has dramatically changed in the last 20 years. Recent series have reported that the overall disease-free survival of patients having no metastasis including the cerebrospinal fluid is 10 years. When extracranial metastases occur, most commonly it involves bone (80%), especially pelvis, long bones, and spine in order of frequency.

## 2. Case Report

**Figure 1:**
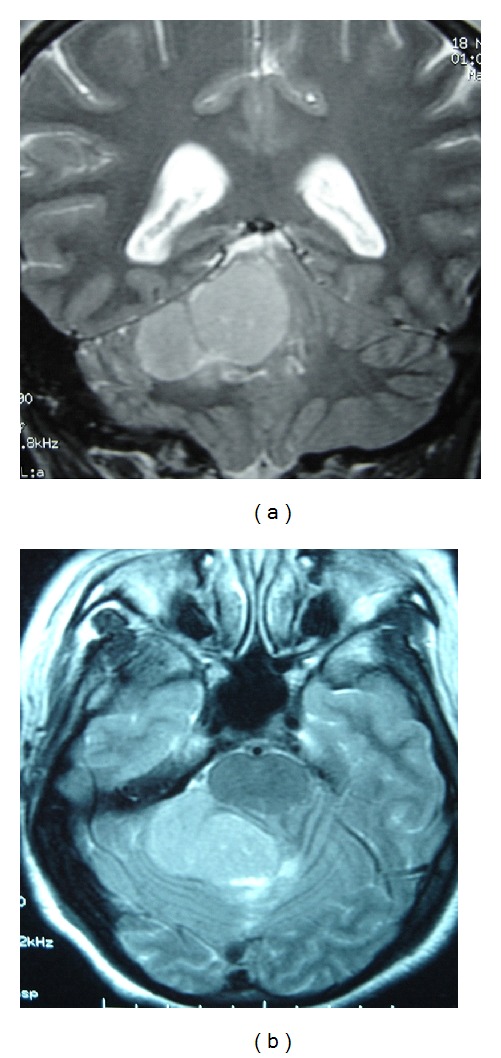
MR showing in T2-weighted hypersignal right cerebellar lesions, with a broad tentorial attachment.

A 34-year-old woman presented with occipital headache of sudden onset, episodic vomiting, and imbalance while walking. Neurologic examination revealed cerebellar syndrome, no sensory or motor deficit. CT and MR of the brain showed a large well-circumscribed, lobulated, heterogeneous space-occupying lesion in the right cerebellar hemisphere measuring 4.1 × 2.5 cm with a broad tentorial attachment (Figure 1). It was predominantly hyperdense on plain CT and had heterogeneous low signal intensity on T1-weighted images, and nonhomogeneous central bright signal areas on T2-weighted images with fairly heterogeneous postcontrast enhancement. The left cerebellar hemisphere and vermis were compressed with a sharp interface between the lesion and brain. Because of its apparent extraaxial location and broad attachment to the tentorium, a working diagnosis of meningioma or medulloblastoma was entertained.

**Figure 2:**
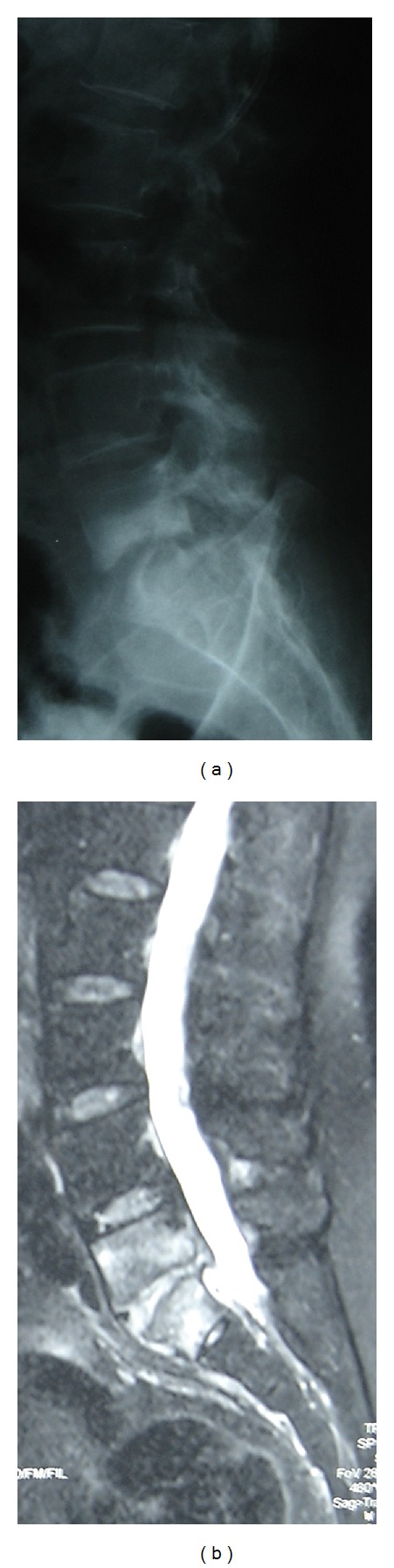
Plain films of the lumbar spine (a) and T2-weighted MR (b) showed diffuse lytic-sclerotic lesions of the vertebral bodies of L5 and S1 and disc lesions mimicking spondylodiscitis.

The patient underwent posterior fossa surgery. Intraoperatively the tumor was soft, easily aspirable with suction and could be clearly separated from the cerebellum. The tentorium was free of tumor, suggestive of an intraaxial lesion. Frozen section revealed malignant small round cells. Histopathological analysis showed nodules of small round cells with occasional neurocytic differentiation and surrounding collagen-rich tissue suggestive of desmoplastic medulloblastoma. Postoperatively, the patient had been given both radiotherapy and chemotherapy. She did well for 3 years with no evidence of recurrence on follow-up CT scans of the brain. She had no other complaints until she developed low back pain and sciatica. Plain radiographs of the lumbar spine showed mixed lytic and sclerotic lesions of the vertebral bodies of L5 and S1 (Figure 2). The described findings presumed spondylodiscitis, and a biopsy of the vertebrae was performed under CT scan guidance. Histopathological examination was reported as primitive neuroectodermal tumor characterized by small, round, and anaplastic cells compatible with medulloblastoma. The patient was hospitalized and underwent a chemotherapy treatment protocol with good outcome for two years. 

## 3. Discussion

Medulloblastoma, or primitive neuroectodermal tumor of the cerebellum, accounts for approximately 20% of all childhood and 1% of all adult brain tumors [1]. It is the third most common central nervous system tumor to exhibit systemic metastases after glioblastoma and meningioma [2, 3]. Most authors agree on a hematogenous route of spread. Spread by shunt tubing has also been described as a consequence of escape of tumor cells into the systemic circulation owing to the break in the blood/brain barrier, which potentially occurs as a result of tumor surgery with the insertion of a cerebrospinal shunt [1, 3–6].

The average interval to the development of extraneural metastasis after the initial diagnosis of medulloblastoma is 18 months. In some cases, 13 years have elapsed after initial diagnosis before metastasis has developed [1, 7, 8]. In our case, the metastasis occurred 2 years later. Long-term survival is possible in adults treated for medulloblastoma. Although rare, metastasis seeding at presentation is a poor prognostic factor. The possibility of delayed recurrence necessitates close followup of all patients. Tumor recurrences should be treated with aggressive therapies as some patients may have sustained response. Adjuvant chemotherapy should be given to high-risk patients, but its role in reducing recurrences, particularly distant ones, remains unclear in the standard-risk group [9, 10].

Bone is the most common site of extraneural spread of medulloblastoma followed by lymph nodes, peritoneum, liver, and lungs [2, 7, 11]. Bone metastases commonly involve the pelvis, long bones, and spine while the ribs and skull are the least affected sites. Chiewvit et al. reported that radiological evidence of bone metastases were twice as frequently blastic as lytic [7]. Mixed patterns are rather seldom (5%) [1]. There are few descriptions about the MR characteristics of the blastic forms, and they present as low signal intensity lesions on both T1- and T2-weighted images [1, 12].

This case is unique in that, to our knowledge, the MRI appearance of the extensive mixed type (both lytic and sclerotic) metastasis in medulloblastoma involving almost the lumbar disc was not described previously. MR appearance of skeletal metastases of medulloblastoma demonstrating the blastic forms has only been described in a limited number of case reports. Diffuse involvement of spine and the disc mimicking spondylodiscitis in the same patient is extremely rare. In our contest, where tuberculosis is a common cause of spondylodiscitis, only the biopsy can help make the diagnosis. In fact, we considered that chemotherapy immunosuppressed this patient and facilitated tuberculous infection. This was the reason for which tuberculous spondylodiscitis was taken into consideration as the first differential diagnosis rather than metastatic medulloblastoma, and this was supported by radioimaging, mimicking vertebral, and lumbar disc infection, that is, the same images found in tuberculosis of the spine.

Although systemic metastases from primary intracranial tumors are uncommon, the onset of bone pain in patients with medulloblastoma should raise suspicion of possible bone involvement. Plain radiographs may be diagnostic, but with the multiplanar capability and high sensitivity in soft tissue contrast, MR images provide better tissue characterization and give detailed anatomic information and extent of the lesions. The MR imaging is more efficient than the bone scintigraphy in detecting vertebral metastasis, especially in the cases that bone scintigraphy is equivocal or negative for vertebral metastasis in high clinical suspicion. Furthermore, MR imaging is important for the further treatment planning such as radiation therapy or systemic chemotherapy. Although MR imaging is useful in the detection of early metastasis that is localized completely in the bone marrow cavity routinely, bone scintigraphy remains the most cost-effective method for examination of the entire skeleton.

In our case, the patient underwent a second-protocol chemotherapy for medulloblastoma treatment with good outcome for two years. The survival results for medulloblastomas in adults compare favorably with those in children. However, late relapses, lateral tumor location, and desmoplastic histologic features are more frequent in adults. Spinal seeding at presentation is a poor prognostic factor for disease-free survival. A minimal dose of 54 Gy to the posterior fossa is essential for adequate tumor control. The interval between surgery and the start of RT, which was found to be a significant prognostic factor, is an interesting issue that requires further study [1, 9, 10].

## 4. Conclusion

Systemic metastases of medulloblastoma occur in a minority of patients. Very rarely such metastases may involve the spine and present with features of spondylodiscitis on imaging. The treatment options for patients with extraneuraxial metastases are limited and their prognosis continues to remain poor.
